# Carotid dissection mimicking a new attack of cluster headache

**DOI:** 10.1186/1129-2377-14-84

**Published:** 2013-10-08

**Authors:** Elisa Candeloro, Isabella Canavero, Maurizia Maurelli, Anna Cavallini, Natascia Ghiotto, Paolo Vitali, Giuseppe Micieli

**Affiliations:** 1 Cerebrovascular Diseases and Stroke Unit, Department of Emergency Neurology, IRCCS National Institute of Neurology Foundation Casimiro Mondino, Pavia, Italy; 2 Neurosonology Unit, Department of Neuropathophysiology, IRCCS National Institute of Neurology Foundation Casimiro Mondino, Pavia, Italy; 3 Department of Neuroradiology, IRCCS National Institute of Neurology Foundation Casimiro Mondino, Pavia, Italy

**Keywords:** Carotid dissection, Cluster headache, Dissection mimics

## Abstract

**Background:**

Symptomatic cluster headache (CH) secondary to internal carotid artery dissection (ICAD) has been frequently reported, however, as far as we know, the coexistence of episodic CH and acute symptomatic CH secondary to ICAD has not.

**Case report:**

A 39 year-old man, affected by episodic CH since the age of 19, presented an atypical headache associated with his usual autonomic symptoms. After a series of negative tests, MRA eventually revealed dissection of the right distal internal carotid artery.

**Discussion and conclusions:**

The coexistence of episodic CH and acute CH symptomatic of ICAD in our patient suggests that, at least in some cases, CH and ICAD may be different expressions of a common underlying cause: hidden vessel wall damage. When risk factors and the change - though partial - of clinical features suggest symptomatic cases, CH patients have to be strictly monitored over time. Given the lack of a *gold standard* investigation for dynamic diseases such as dissections, these patients require multimodal diagnostic investigation over time, even in cases where exams are normal at onset.

## Background

It is widely recognized that carotid dissection could simulate a cluster headache (CH) attack. In fact, many cases of symptomatic CH secondary to internal carotid artery dissection (ICAD) have been reported [1-5]. However, to our knowledge, the occurrence of acute symptomatic CH secondary to ICAD in a patient affected by episodic CH has never been reported. We speculate about a pathogenetic connection between the two conditions in our patient. The peculiarity of the case also offers an example of a challenging differential diagnosis in the emergency department setting.

## Case presentation

On 8th March 2012, a 39 year-old man presented to the emergency department 12 hours after the onset of right orbital, enduring, pressing pain. Neurological examination revealed miosis and ptosis in the right eye.

From the age of 19 the patient had suffered from episodic cluster headache, with attacks of right-orbital boring pain, lasting about 30 minutes, with conjunctival injection, rhinorrea, miosis and ptosis in the right eye. The condition was responsive to sumatriptan and verapamil. His last cluster attack had occurred in May 2011. In previous years he had undergone several neuroimaging examinations (computed tomography, magnetic resonance imaging, magnetic resonance angiography), showing no pathological findings. Past medical history also revealed a childhood post-traumatic fracture of the right petrous apex; MTHFR C677T homozygosis. The patient was practicing sport intensively, including the use of fitness equipment that implied the repetitive flexion-extension of the neck.

**Figure 1:**
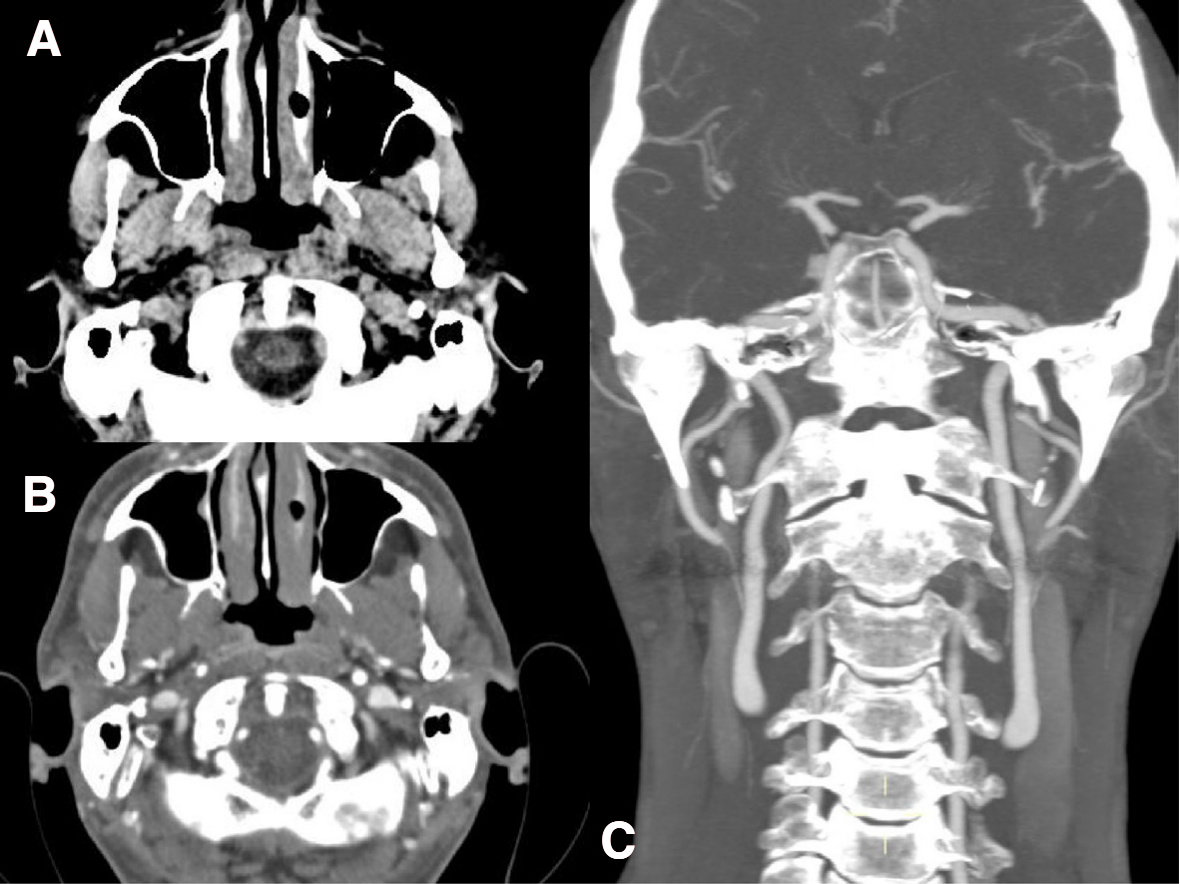
**CT findings (performed in emergency department). A**. Non-contrast axial CT shows normal findings. **B**. Axial CTA shows only a subtle asymmetry of the internal carotid lumen filling by contrast and was reported as negative for artery dissection. **C**. Coronal CTA shows normal findings.

The atypically enduring pain led us to exclude secondary forms of cluster headache. An urgent brain computed tomography (CT) with computed tomography angiography (CTA) of intra-extracranial vessels was performed in the emergency department. CTA showed only a subtle asymmetry of the internal carotid lumen filling by contrast (Figure 1A, 1B) and was reported as negative for artery dissection.

**Figure 2:**
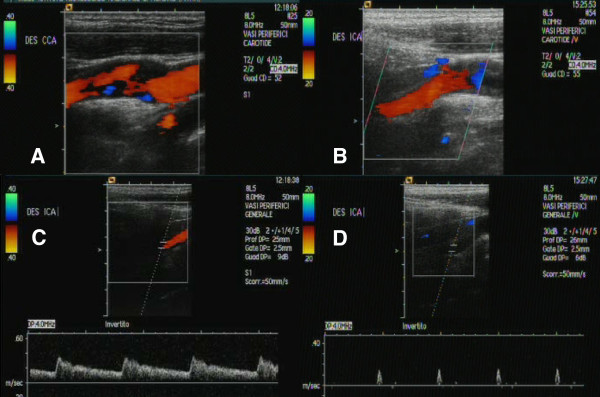
**Duplex sonography findings. A**. B-mode image performed on 9th March: complete filling of the right ICA lumen with normal PRF. **B**. B-mode image performed on 14th March: complete filling of the right ICA lumen with normal PRF. **C**. Doppler flow measurement performed on 9th March: normal waveform. **D**. Doppler flow measurement performed on 14th March: a waveform with a very low amplitude, high-resistance and no diastolic flow.

**Figure 3:**
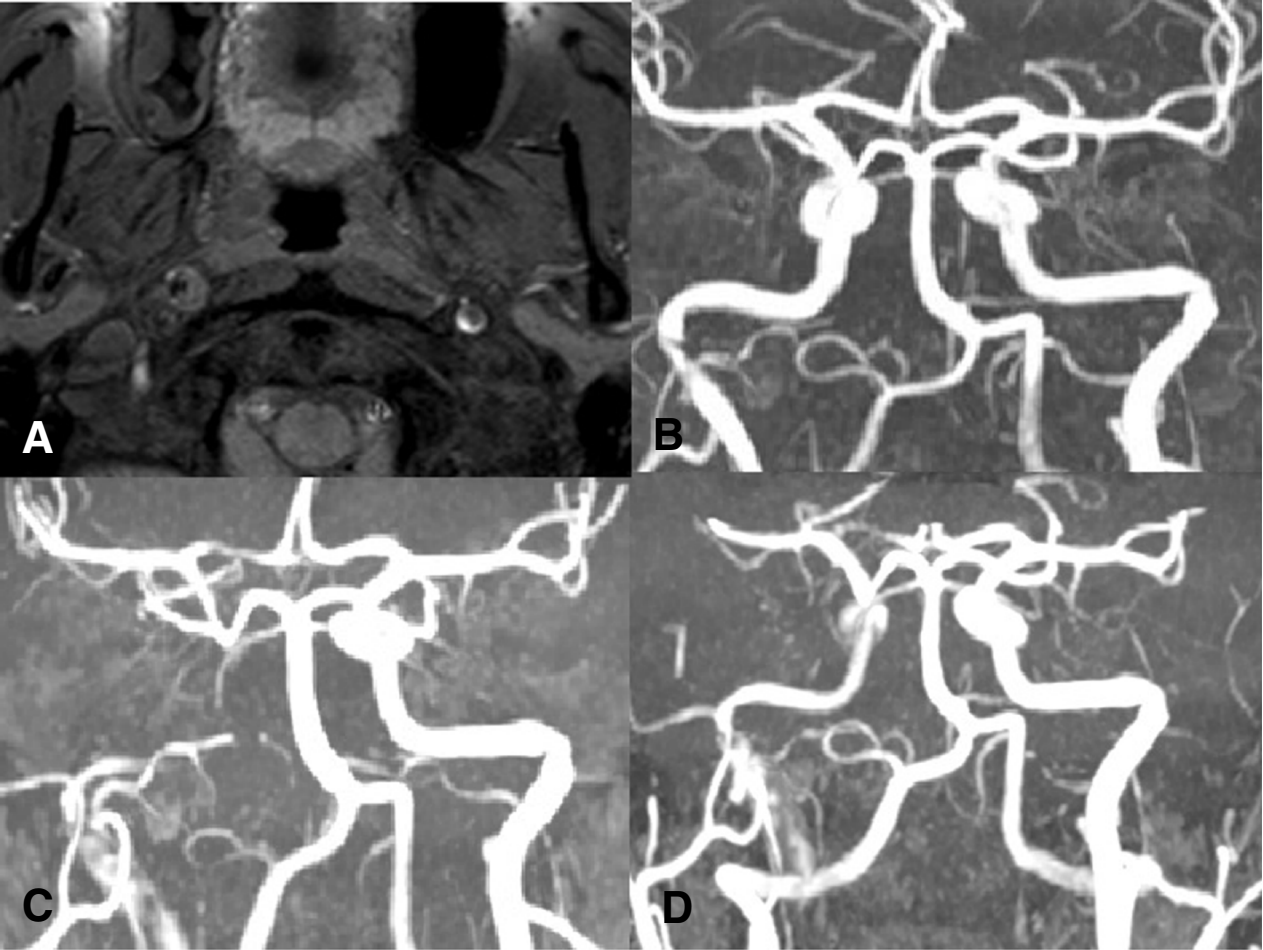
**MR findings. A**. Axial T1 fat-sat image (performed on 9th March) shows the typical semilunar image of the intramural hematoma in the right ICA. **B**. Time-of-flight (TOF) MRA (performed on 9th March) shows a focal “minus” image at the extra-intracranial passage in the right ICA. **C**. On 14th March MRA shows extension of contrastographic defect in the right ICA, due to the cranio-caudal extension of the intramural hematoma. **D**. On 19th March MRA shows a subtotal re-canalisation of the right ICA.

Due to persisting pain and Horner’s syndrome, the man was admitted to the neurology department for further investigations. On 9th March he underwent a Duplex sonography of the supra-aortic vessels and a transcranial Doppler; the findings were normal (complete filling of the ICA lumen with normal Pulse Repetition Frequency [Figure 2A] and a normal waveform [Figure 2C]). A few hours later, a brain magnetic resonance imaging (MRI) with an extra-intracranial magnetic resonance angiography (MRA) demonstrated an intramural hematoma of the right distal internal carotid (about one centimeter before the intracranial segment), without stenosis of the vessel (Figure 3A, 3B). He was immediately anti-coagulated with heparin in order to prevent a stroke.

During hospitalization, the patient developed arterial hypertension, bradycardia and transient paresthesias of the right hemiface. For this reason, on the 5th day Duplex sonography was performed and disclosed a steno-occlusive distal process: the investigation disclosed the complete filling of the lumen with Pulse Repetition Frequency (Figure 2B) and a waveform with a very low amplitude, high-resistance and no diastolic flow (Figure 2D); the increased pulsatility and notable reduction in blood flow amplitude and velocity were consistent with the development of a stenosis downstream and suggested a possible worsening of carotid dissection. An extra-intracranial MRA showed apparent carotid occlusion (Figure 3C) and DWI sequences on MRI and ruled out the occurrence of a stroke. On the 10th day subtotal carotid recanalization was documented by MRA (Figure 3D). At the six-month follow up, neurological examination was normal and neuroimaging showed stable partial recanalization.

## Discussion

To our knowledge, a coexistence of episodic CH and acute symptomatic CH secondary to ICAD has never been previously reported.

CH and ICAD could present with unilateral orbital pain in association with ipsilateral miosis and ptosis due to sympathetic dysfunction. Either in CH or in ICAD, rarely, it could be found also unilateral tearing, conjunctival injection and rhinorrhea due to parasympathetic dysfunction [4,5].

In both CH and ICAD, the pericarotid plexus is involved in autonomic symptoms and trigeminal fibers in pain and parasympathetic symptoms [1,4].

According to some authors, in CH the simultaneity of pain and autonomic symptoms is due to a “pathophysiologic focus” in the superior pericarotid cavernous sinus plexus [6], where the fibers of the trigeminal nerve, superior cervical ganglion and sphenopalatine ganglion take connection [1-7]. Evidence for such a “peripheral” origin of symptoms comes from the observation that clinical features of symptomatic forms secondary to the involvement of these structures are almost indistinguishable from those in primary CH [8].

In ICAD, local signs and symptoms are thought to derive from the involvement of the adjacent structures. Autonomic symptoms could derive from the stretching and disruption of the pericarotid sympathetic nerve fibers, due to the enlargement of the vessel by intramural hematoma [9]. Pain could be determined by stimulation of the trigemino-vascular system [3]. An enlarged vessel can also determine the compression of other contiguous structures such as the carotid sinus and the trigeminal nerve. In our patient, the compression of these structures resulted in “baroceptor failure syndrome”, with hypertension and bradycardia, and transient parestesias in the right hemiface.

In ICAD pathogenesis, it has been hypothesized that an interaction between genetic (e.g. MTHFR C677T homozygosis) and environmental factors (e.g. cranio-cervical traumas, mechanical stresses during sports activity and chiropractic manipulation) may lead to the initial vessel wall damage [10].

Therefore, in our case, the presence of several of these risk factors (the post-traumatic petrous apex fracture, MTHFR C677T homozygosis and repeated mechanical stress during sports activity), the coexistence of CH and ICAD with overlapping clinical features and the inferable involvement of the same afore-mentioned anatomical structures, suggest that the two diseases could share a common “pathophysiologic focus”: probably hidden vessel wall damage.

A clinical differential diagnosis between CH and ICAD is influenced by the similarity of the symptoms, even more so if the two conditions co-exist. In ICAD pain is often associated with ipsilateral Horner syndrome, while other autonomic symptoms are unusual. However, in literature it has been described a case of ICAD whose CH-like symptoms were Horner syndrome, tearing and rhinorrhea [4]. The time course of pain was the only feature to be distinctive from CH, as in our patient.

Clinical observation and monitoring are crucial in order to note every new, atypical feature of symptoms such as, in our case, enduring pain.

However, anamnestic and clinical data could be unable to support the differential diagnosis, as reported by Godeiro-Junior et al. [5]: in their report ICAD presented with CH-like pain, Horner syndrome and other autonomic symptoms. For these reasons, further investigations are mandatory.

Nevertheless, even instrumental differential diagnosis is hindered by some factors: the heterogeneous anatomopathological features of vessel wall injury, the high dynamicity of dissections, and the intrinsic limits of the available instrumental techniques (especially in emergency departments).

Some Authors have proposed that spontaneous cervical artery dissections affect primarily the outer arterial layers, taking origin from a degenerative process at the medial-adventitial border. This may lead to the formation of a neoangiogenetic network and, subsequently, intramural hematomas [11]. The direction of expansion of the intramural hematoma determines the successive steps: if it is towards the lumen, stenosis or occlusion of the vessel could occur, with, though not necessarily, the formation of a double-lumen and an intimal flap; if it is towards the surrounding tissues, it could produce dilatation of the vessel and cause compressive local signs. Intimal flap, double lumen, pseudoaneurysm are considered pathognomonic of arterial dissection, but not invariably found. Their absence does not exclude the diagnosis. Further, it is difficult to predict if dissection will evolve into arterial steno-occlusion or recanalization; the timing of these changes could be even harder to predict [12]. It follows that the assumption that there is a correspondence between the time of the onset of dissection and the time of the onset of symptoms and signs may not be true in all cases [13], as the incidental finding of asymptomatic cases demonstrates.

The high anatomical heterogeneity and the unpredictable natural course of ICAD hinder instrumental diagnoses, especially in the absence of a *gold standard* test [13]. The available techniques are able to evaluate different aspects of the disease. CTA investigates mainly the lumen; it has a good sensitivity for stenosis, intimal flap and pseudoaneurysm but poor sensitivity for isolated intramural hematoma [14]. Owing to the mostly distal location of ICAD, often only indirect signs are detectable with Duplex sonography. This technique assesses a reduction of blood flow with good sensitivity only with stenosis > 50% downstream; ultrasound investigation has poor sensitivity in non-stroke patients with isolated Horner syndrome [15]. B-mode images visualize the arterial wall and the surrounding tissue: they may identify the double lumen but with poor sensitivity and only when located in the proximal extracranial carotid segment [16]. MRA evaluates the vessel wall and is able to detect intramural hematoma despite vessel occlusion: axial MRA T1-weighted imaging with fat suppression allows an optimal discrimination between intramural hematoma and perivascular tissue [17].

At the onset of symptoms in our patient the only sign of artery dissection was an isolated intramural hematoma, as disclosed by MRA; CTA was reported as normal given the absence of stenosis/intimal flap/pseudoaneurysm and Duplex sonography was normal due to the distal non-stenosing localization of the injury. Over the following days, ultrasounds showed abnormal findings due to the development of a significant stenosis that was confirmed by MRA.

## Conclusions

Our case suggests that, in some cases, episodic CH and symptomatic CH in ICAD could share a common etiopathogenetic mechanism. This being, to our knowledge, the first case of association between CH and ICAD, and considering the available current literature [8,13], we believe that this hypothesis deserves attention and requires further studies to be confirmed.

From this report we could infer that patients with typical CH attacks [18], in the presence of risk factors for vessel wall damage, should undergo a careful clinical-instrumental follow up, paying particular attention to the development of atypical clinical features, such as pain.

This case highlights the need to combine different investigations and repeat them over time in CH patients who develop new atypical symptoms suggestive of ICAD. In fact, a negative initial investigation does not rule out the diagnosis of dissection.

## Consent

Written informed consent was obtained from the patient for publication of this Case report and the accompanying images. A copy of the written consent is available for review by the Editor-in-Chief of this journal.

## Abbreviations

CH: Cluster headache; ICA: Internal carotid artery; ICAD: Internal carotid artery dissection; MRI: Magnetic resonance imaging; MRA: Magnetic resonance angiography; CT: Computed tomography; CTA: Computed tomography angiography; PRF: Pulse repetition frequency.

## Competing interests

The authors declare that they have no competing interests.

## Authors’ contributions

CE conceived of the study, participated in clinical management of the patient, reviewed the literature on the item and drafted the manuscript. CI participated in the design of the paper, in reviewing the literature and drafting the manuscript. MM had the first approach with the patient at the ER, made the correct diagnosis and participated in his clinical management. CA has contributed in the clinical management of the patient and revised the manuscript. GN performed the neurosonological investigation and reviewed the literature concerning the technique. VP performed the neuroradiological investigation and reviewed the literature concerning the technique. MG has contributed in the clinical management of the patient and revised the manuscript. All authors read and approved the final manuscript.
